# Supplementary material for: On the generalizability of diffusion MRI signal representations across acquisition parameters, sequences and tissue types: Chronicles of the MEMENTO challenge
Source: Neuroimage. Author manuscript; Available in PMC 2023 Oct 28. (PMC7615259; doi:10.1016/j.neuroimage.2021.118367)
Supplement: Appendix [file EMS189821-supplement-Appendix.pdf]

Supplementary materials

Supplementary material associated with this article can be found, in the online version, at doi:10.1016/j.neuroimage.2021.118367.

Appendix A

A.1. Tensor-based models

- DTI: The diffusion tensor imaging method was fitted with a linear least squares procedure to determine the diffusion tensor (6 parameters) and the average non-weighted signal (1 parameter).
- DKI: The diffusion kurtosis imaging extends the DTI method to account for restricted diffusion. It was fitted with a weighted least squares procedure using ExploreDTI to determine 22 parameters: 6 for the diffusion tensor, 15 for the kurtosis tensor and the non-weighted signal. No additional constraints were considered in this fit.
- DKI+Offset: The DKI model was extended to accommodate an additional degree of freedom modelling a positive constant bias in the signal due to, for example, Rician noise. The 23 free parameters of this model were fitted with a non-linear least squares procedure implemented in MATLAB, constraining a monotonic signal decay and enforcing both the diffusion and kurtosis tensor to be positive definite.
- DTD-cov: The diffusion tensor distribution (DTD) method describes the diffusion signal as the sum of a distribution of microscopic tensors. The 28 parameters of the fourth order covariance tensor method were fitted to the data with a non-linear least squares procedure implemented in MATLAB, constraining a monotonic signal decay and enforcing both the diffusion and kurtosis tensor to be positive definite.
- DTD-cov+Offset: The DTD-cov method was extended with one additional degree of freedom modelling a positive constant bias in the signal due to, for example, Rician noise. The 29 free parameters of this model were fit with a non-linear least squares procedure implemented in MATLAB, constraining a monotonic signal decay and enforcing both the diffusion and kurtosis tensor to be positived definite.

Multi-compartment models

- Ball&Stick: originally proposed from Behrens and colleagues, this model consists of two compartments: a stick (impermeable cylinder with zero radius) to model anisotropic restricted intra-cellular diffusion, and a ball to model isotropic hindered extra-cellular diffusion. The model was implemented in Python using the Dmipy package, and its 4 parameters fitted to the data using a two stages procedure consisting of an initial grid search, followed by a constrained non-linear fit procedure based on a limited-memory quasi-Newton method.
- Ball&Racket: this model is an extension of the Ball&Stick that explicitly takes into account fanning configurations. The 7 parameters of the model were fitted to the data using the same procedure described for the Ball&Stick model.
- NODDI-Watson: originally introduce from Zhang et al., this model accounts for intra-cellular diffusion modelled as a tensor convolved with a Watson distribution to account for axonal dispersion, an extracellular compartment modelled with a Zeppelin, and an isotropic free water component to account for partial volume with the cerebrospinal fluid. The volumes of the intracellular and extracellular compartments are linked with a tortuosity principle, and the parallel diffusivity of the tensor is set to $1.7 \times 10^{-3}\mathrm{mm}^2/\mathrm{s}$. The 5 parameters of the model were fitted to the data using the same procedure described for the Ball&Stick model.
- NODDI-Bingham: this model extends the NODDI-Watson model to account for asymmetric fiber dispersion using a Bingham distribution. The 7 parameters of the model were fitted to the data using the same procedure described for the Ball&Stick model.
- SMT: The spherical mean technique (SMT) model provides estimates of neurite density and of the intrinsic tissue diffusivity unconfounded by fibre crossings and orientation dispersion. The 51 parameters of the model were fitted to the data using the same procedure described for the Ball&Stick model.
- NODDI-SMT: This is a reformulation of the NODDI-Watson model using the SMT technique. The 50 parameters of the model were fitted to the data using the same procedure described for the Ball&Stick model.
- MCMDI: this model describes intra-cellular diffusion with a stick, and extra-cellular diffusion with a Zeppelin. The SMT technique is used to achieve invariance to fibre crossing and orientation dispersion. The 50 parameters of the model were fitted to the data using the same procedure described for the Ball&Stick model.
- ActiveAx: introduced from Dyrby and colleagues, this model describes intra-cellular diffusion as a cylinder with finite radius, extracellular diffusion as a zeppelin, and accounts for isotropic contamination due to cerebrospinal fluid. The 7 parameters of the model were fitted to the data using the same procedure described for the Ball&Stick model.

A.3. Parametric representations

- SHORE: The method is based on the original simple harmonic oscillator reconstruction (SHORE). SHORE with optimized reconstruction was tested at different orders of 6, 8 and up to 12. However, the best results or lower errors were determined to be at either order 6 or 8. The 50 parameters of the model were fitted to the data using a linear least-squares approach.
- MAP-MRI: Mean Apparent Propagator Magnetic Resonance Imaging (MAP-MRI) is a linear representation of the diffusion signal that uses a 3D generalization of the SHORE basis. The 95 parameters of the method were fitted using a penalized least-squares procedure with generalized cross validation implemented.
- MAP-MRI+Reg: This submission used the Laplacian-regularized MAP-MRI method of order 8 implemented in the Dipy software library with no positivity constraint in the propagator and a regularization weight of 0.47 to fit the 95 free parameters of the method.

A.4. Deep-learning methods

- NeuralNet: A fully connected neural network with a single hidden layer of 50 neurons and using sigmoid activation functions was trained to predict the unprovided signal amplitudes for each measurement independently. The 50 parameters of the network were optimized based on the mean squared error of the predictions us-

Table A1
The valid signal predictions submitted to the MEMENTO challenge. For each method, we report the acronym and the main reference, the "category", special notes on the fit procedure, and the data it has been applied to. The following predictions were subdivided in the following categories: tensor-based (TENS), multi-compartment model (MCM), parametric representation (PAR), deep learning-based (DL).

Model name	Category	Implementation details	Computation time [voxel]	Number of free parameters	Noise assumptions	Optimization algorithm
DTI (Basser et al., 1994)	TENS	-	< 1s	7	Gaussian	Linear Least Squares
DKI (J. H. (Jensen et al., 2005)	TENS	Implemented in ExploreDTI	< 1s	22	Gaussian	Weighted Least Squares
DKI+Offset (Morez et al., 2020)	TENS	Monotonic signal decay, positive definite tensor	< 1s	23	Rician	Non-linear least-squares
DTD-cov (C. F. (Westin et al., 2016)	TENS	Monotonic signal decay, positive definite tensor	< 1s	28	Gaussian	Non-linear least-squares
DTD-cov (C. F. (Westin et al., 2016) + Offset	TENS	Monotonic signal decay, positive definite tensor	< 1s	29	Rician	Non-linear least-squares
Ball&Stick (Behrens et al., 2003)	MCM	Implemented in Dmipy (Fick et al., 2019)	< 1s	4	Gaussian	Constrained non-linear least-squares
Ball&Racket (Sotiropoulos et al., 2012)	MCM	Implemented in Dmipy (Fick et al., 2019)	< 1s	7	Gaussian	Constrained non-linear least-squares
NODDI-Watson (Zhang et al., 2012)	MCM	Implemented in Dmipy (Fick et al., 2019)	< 1s	5	Gaussian	Constrained non-linear least-squares
NODDI-Bingham (Tariq et al., 2016)	MCM	Implemented in Dmipy (Fick et al., 2019)	< 1s	7	Gaussian	Constrained non-linear least-squares
SMT (Kaden et al., 2016)	MCM	Implemented in Dmipy (Fick et al., 2019)	< 1s	51	Gaussian	Constrained non-linear least-squares
NODDI-SMT	MCM	Implemented in Dmipy (Fick et al., 2019)	< 1s	50	Gaussian	Constrained non-linear least-squares
MCMDI (Kaden et al., 2016)	MCM	Implemented in Dmipy (Fick et al., 2019)	< 1s	50	Gaussian	Constrained non-linear least-squares
ActiveAx (D. C. (Alexander et al., 2010)	MCM	Implemented in Dmipy (Fick et al., 2019)	< 1s	7	Gaussian	Constrained non-linear least-squares
SHORE (Ozarslan et al., 2009)	PAR	From DeepSHORE (Nath et al., 2019)	< 1s	50	Gaussian	Regularized least squares
MAP-MRI Ozarslan et al., 2013	PAR		< 1s	95	Gaussian	Constrained quadratic programming
MAP-MRI+Reg (Fick et al., 2016)	PAR	Implemented in Dipy (Garyfallidis et al., 2014)	33s	95	Gaussian	Regularized least squares
NeuralNet	DL	Perceptron 1 Layer 50 nodes	Training: ~ 70s, Prediction: <1s	Signal dependent	Gaussian	Adam
NeuralNet+Reinf (Williams, 1992)	DL	Perceptron 7 Layers optimized with NAS (Zoph and Le, 2016)	NA	Up to 896	Gaussian	Adam

ing the ADAM algorithm with a learning rate of 0.005 over 20000 epochs. For SDE-MS and SDE-GRID the normalized components of the gradient (3 values) and the b-value were provided as inputs to the network. For the DDE and DODE acquisitions, the gradient strength, the normalized components of the two gradients (6 values), the b-value, and the components of the b-matrix (6 values) were concatenated into one input vector of length 14.

- NeuralNet+Reinf: A fully connected neural network with reinforcement learning. The authors adopted a neural architecture search (NAS) to identify the optimal 7-layer perceptron model for dMRI signal prediction with either 8. 16, 32, 64 or 128 nodes per layer. The free parameters of the network ranged between 56 and 896, and their values was optimized with the ADAM method using an initial learning rate equal to 0.01 and 200 training epochs.

Table A1.
